# Induction of oxidative stress causes functional alterations in mouse urothelium via a TRPM8-mediated mechanism: implications for aging

**DOI:** 10.1111/acel.12208

**Published:** 2014-03-05

**Authors:** Linda Nocchi, Donna M Daly, Christopher Chapple, David Grundy

**Affiliations:** 1 Department of Biomedical Science, University of Sheffield, Western BankSheffield, S10 2TN, UK; 2 Department of Urology, Royal Hallamshire HospitalGlossop Road, Sheffield, S10 2JF, UK

**Keywords:** aging, mouse, oxidative stress, reactive oxygen species

## Abstract

The incidence of bladder conditions such as overactive bladder syndrome and its associated urinary incontinence is highly prevalent in the elderly. However, the mechanisms underlying these disorders are unclear. Studies suggest that the urothelium forms a ‘sensory network’ with the underlying innervation, alterations in which, could compromise bladder function. As the accumulation of reactive oxygen species can cause functional alterations with age, the aim of this study was to investigate whether oxidative stress alters urothelial sensory signalling and whether the mechanism underlying the effect of oxidative stress on the urothelium plays a role in aging. Five-month-old(young) and 24-month-old (aged) mice were used. H_2_O_2_, used to induce oxidative stress, resulted in an increase in bladder afferent nerve activity and urothelial intracellular calcium in preparations from young mice. These functional changes were concurrent with upregulation of TRPM8 in the urothelium. Moreover, application of a TRPM8 antagonist significantly attenuated the H_2_O_2_-induced calcium responses. Interestingly, an upregulation of TRPM8 was also found in the urothelium from aged mice, where high oxidative stress levels were observed, together with a greater calcium response to the TRPM8 agonist WS12. Furthermore, these calcium responses were attenuated by pretreatment with the antioxidant *N*-acetyl-cysteine. This study shows that oxidative stress affects urothelial function involving a TRPM8-mediated mechanism and these effects may have important implications for aging. These data provide an insight into the possible mechanisms by which oxidative stress causes physiological alterations in the bladder, which may also occur in other organs susceptible to aging.

## Introduction

The lower urinary tract and its innervation appear to be particularly susceptible to aging such that the incidence of bladder-related symptoms, as seen in overactive bladder syndrome (OAB) and in particular urinary incontinence (UI), increases in the elderly; however, the aetiology of these bladder disorders is still not defined. The inner lining of the bladder known as the urothelium was initially considered to be a passive barrier between the luminal contents and the underlying tissues. However, studies now suggest that in addition to its barrier function, the urothelium is a dynamic structure that actively contributes to the sensory function of the bladder via the release of excitatory and inhibitory mediators in response to mechanical and chemical stimuli. The urothelium interacts closely with the underlying sensory innervation, and together these structures form a functional ‘sensory web’, which has been purported to play an important role in normal bladder function. Studies suggest that alterations to urothelial structure and compromised urothelial sensory signalling could underlie several bladder disorders (Birder *et al*., 2012).

The widely accepted oxidative stress theory of aging states that accumulation of reactive oxygen species (ROS) occurs with age and leads to functional alterations and pathological conditions (Kregel & Zhang, 2007). Experimental studies in rodents suggest that aging impairs contractile response in the bladder, and this effect correlates with an increase in oxidative stress markers, suggesting that oxidative stress may contribute to alter bladder function in aging (Gomez-Pinilla *et al*., 2007b). The hypothesis that an age-related accumulation of ROS can affect bladder function is strengthened by several studies showing that treatment with antioxidants, such as melatonin and green tea catechins, can improve bladder physiology (Gomez-Pinilla *et al*., 2007a,b, 2008; Juan *et al*., 2012). However, the mechanisms underlying the effect of oxidative stress on bladder function are still unclear.

A number of studies have demonstrated that several transient receptor potential (TRP) channels are regulated by oxidative stress and that calcium influx through TRP channels may be one mechanism by which oxidative stress mediates cell damage and physiological alterations (Miller, 2006; Poteser *et al*., 2006; Miller & Zhang, 2011; Takahashi *et al*., 2011). Previous studies have suggested a role for the cold sensing TRP melastatin 8 ion channel (TRPM8) in bladder sensation, showing a positive correlation between TRPM8 expression and voiding frequency in patients with bladder conditions (Mukerji *et al*., 2006b) and an involvement of this channel on micturition reflexes and nociceptive signalling (Lashinger *et al*., 2008). Furthermore, intravesical instillation of cold saline into the bladder, during a diagnostic ice water test, can evoke a response, with or without pain in patients with OAB or painful bladder syndrome (Mukerji *et al*., 2006a). It has been postulated that this response could be mediated by TRPM8. However, the pathway by which TRPM8 contributes to bladder dysfunction is still unclear.

The aim of this study was to investigate whether oxidative stress alters urothelial sensory signalling in the bladder and whether the mechanism underlying the effect of oxidative stress on the urothelium plays a role in aging. Although this article looks at the oxidative stress effects specifically on the urothelium, the activation of specific signalling pathways triggered by a rise in intracellular oxidant levels may affect numerous cellular processes linked to aging and lead to the development of age-related diseases in many other organs.

## Results

### Induction of oxidative stress using H_2_O_2_ generates an increase in bladder afferent nerve activity in young mice

**Figure 1:**
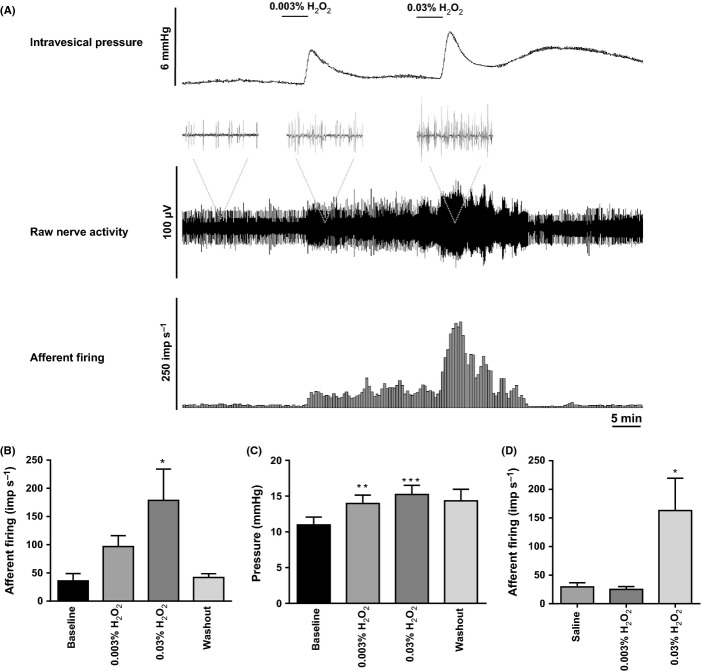
*In vitro* extracellular nerve recording. Application of H_2_O_2_ causes an increase in bladder afferent activity in young mice. (A) Representative trace showing changes in intravesical pressure and bladder afferent nerve activity following bath application of 0.003% and 0.03% H_2_O_2_. (B) Representation of changes in nerve firing in response to bath application of 0.003% and 0.03% H_2_O_2_ compared with baseline (*N* = 6; one-way ANOVA followed by Dunnet’s *post hoc* test, *P < 0.05 significant by Dunnet’s *post hoc* test). (C) Representation of changes in intravesical pressure following extravesical application of 0.003% and 0.03% H_2_O_2_ (*N* = 6; one-way ANOVA followed by Dunnet’s *post hoc* test, **P < 0.001, ***P < 0.0005, significant by Dunnet’s *post hoc* test). (D) Representation of changes in nerve firing in response to intravesical perfusion of 0.003% and 0.03% H_2_O_2_ compared with saline perfusion (*N* = 6; one-way ANOVA followed by Dunnet’s *post hoc* test, *P < 0.05 significant by Dunnet’s *post hoc* test).

To investigate whether induction of oxidative stress alters bladder afferent nerve activity, we performed *in vitro* extracellular nerve recordings in response to extra- and intravesical perfusion of two cumulative concentrations of H_2_O_2_ 0.003% and 0.03% in preparations from young mice (*N* = 6; see Collins *et al*., 2013 for experimental set-up; Collins *et al*., 2013). As shown in Fig. 1B, a dose-dependent increase in nerve firing was found when H_2_O_2_ was applied extravesically under isovolumetric conditions (15 mmHg); however, only the highest concentration of H_2_O_2_ generated a statistical significant increase in nerve activity (one-way ANOVA, Dunnet’s *post hoc* test, *P < 0.05). A moderate increase in bladder pressure was also observed (4–5 mmHg), suggesting that H_2_O_2_ induced muscle contraction (Fig. 1C, one-way ANOVA, Dunnet’s *post hoc* test, **P < 0.001 and ***P < 0.0005). Strikingly, the afferent response was recoverable after only a few minutes, but the changes in pressure were not readily reversible. As shown in Fig. 1D, intravesical application of the lowest concentration of H_2_O_2_ 0.003% did not cause any effect on afferent nerve activity, whereas the highest concentration 0.03% generated a significant increase in firing (Fig. 1D, one-way ANOVA, Dunnet’s *post hoc* test, *P < 0.05). The different effects on the nerve activity observed when the lowest concentration (0.003%) was applied extra- and intravesically are likely to reflect the barrier function of the urothelium, which prevents luminal agents from crossing through the bladder wall.

### Induction of oxidative stress using H_2_O_2_ causes an increase in intracellular calcium in urothelial cells isolated from young mice

**Figure 2:**
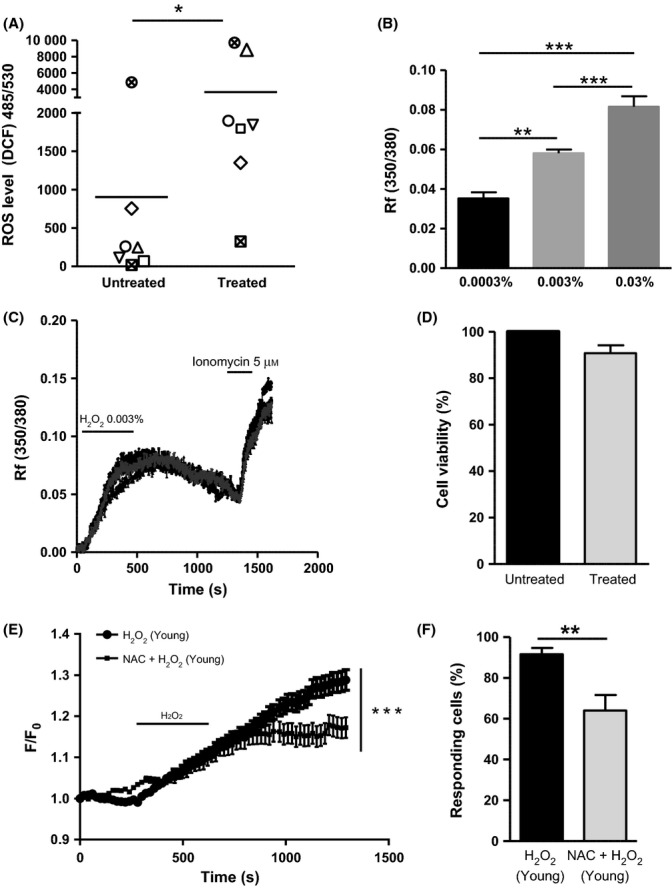
Induction of acute oxidative stress using H_2_O_2_ in the urothelial cells from young mice causes increase in intracellular calcium, which is attenuated by a pretreatment with the antioxidant *N*-acetyl-cysteine (NAC). (A) Detection of intracellular reactive oxygen species (ROS) level using DCF probe showing an increase in ROS level after exposure of the urothelial cells to 10 min 0.003% H_2_O_2_ (*N* = 7; Students *T*-test, *P = 0.04). (B) Dose-dependent increase in intracellular calcium in response to three different concentrations of H_2_O_2_ (0.0003%, 0.003% and 0.03%, one-way ANOVA followed by Bonferroni *post hoc* test; **P < 0.05 and ***P < 0.001 significant by Bonferroni). (C) Representative traces showing changes in urothelial intracellular calcium following 10 min 0.003% H_2_O_2_ and 3 min 5 μm ionomycin application. (D) MTT cell viability assay shows no change in urothelial cell viability following 10 min 0.003% H_2_O_2_ treatment (*N* = 3). (E) Calcium imaging data show an increase in intracellular calcium in the urothelial cells after induction of oxidative stress (*N* = 5, *n* = 169). The response is significantly attenuated by 1 h preincubation with the antioxidant NAC (*N* = 4, *n* = 117; two-way ANOVA, ***P < 0.0001). (F) Percentage of responding cells to H_2_O_2_ ± NAC (Student’s *t*-test, **P = 0.008).

Application of 0.003% H_2_O_2_ for 10 min in urothelial cells isolated from young mice significantly induced oxidative stress as an increased in intracellular ROS was observed (Fig. 2A, *N* = 7, Student’s *t*-test, *P = 0.04). To investigate whether the induction of oxidative stress alters urothelial function, calcium imaging experiments were performed on urothelial cells in response to H_2_O_2_. A dose-dependent increase in intracellular calcium was observed (0.0003–0.03%, Fig. 2B, one-way ANOVA followed by Bonferroni *post hoc* test, ** and *** represent *P* < 0.05 by Bonferroni) and 0.003% was used in subsequent experiments. Figure 2C shows representative traces of the calcium response profile of four cells (*n* = 4), following a 10 min perfusion of 0.003% H_2_O_2_ and 3 min perfusion of the ionophore ionomycin (5 μm). Cell viability was assessed using the MTT assay, and no change in cell viability was observed after 10 min application of 0.003% H_2_O_2_ (Fig. 2D, *N* = 3, Student’s *t*-test). The *N*-acetyl-cysteine (NAC) was used as antioxidant as it has been previously shown to act as ROS scavenger by replenishing intracellular glutathione stores (Small *et al*., 2012). Pretreatment with 10 mm NAC (1 h) significantly attenuated the calcium response and reduced the percentage of cells responding to H_2_O_2_ (*N* = 4, *n* = 117 and *N* = 5, *n* = 169, respectively, Fig. 2E,F, two-way ANOVA, ***P < 0.0001).

### TRPM8 expression is upregulated by oxidative stress and is involved in oxidative stress-induced calcium mobilization in the urothelium

**Figure 3:**
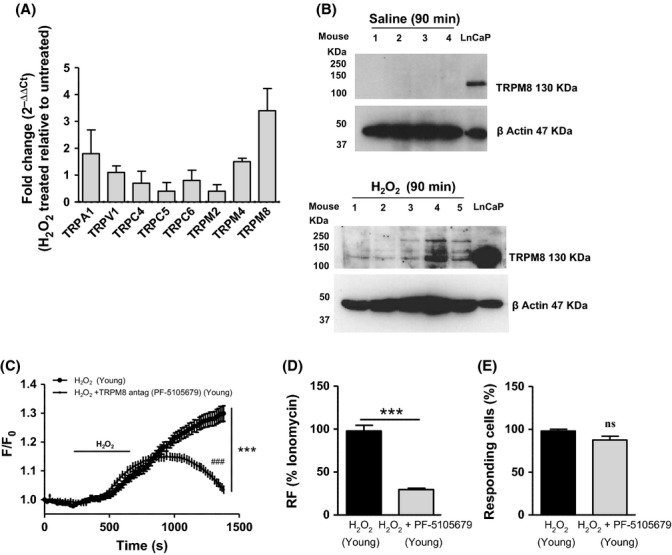
Induction of oxidative stress in the urothelium from young mice causes an upregulation of TRPM8 that mediates H_2_O_2_-induced calcium response. (A) qRT-PCR for TRPA1, TRPV1, TRPC4, TRPC5, TRPC6, TRPM2, TRPM4 and TRPM8 mRNA expression in urothelium samples following 5 h exposure to 0.003% H_2_O_2_. Results are expressed as fold change (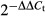) of treated compared with the untreated group, and TRPM8 shows the greatest upregulation with a 3.4-fold change in the H_2_O_2_ treated group. (B) Western blot for TRPM8 protein level in the urothelium dissected from the bladder after 90 min intravesical perfusion with saline (*N* = 4; lanes 1–4 represent different mice) or H_2_O_2_ (*N* = 5; lanes 1–5 represent different mice). Results show an upregulation of TRPM8 protein following H_2_O_2_ treatment. LNCaP prostate cancer cell line was used as TRPM8-positive control to assess the right band size, and β-actin was used as loading control. (C) Calcium imaging data show an attenuation of H_2_O_2_-induced calcium response in the presence of the TRPM8 antagonist PF-5105679 in urothelial cells from control mice (*N* = 4, *n* = 224, *n* = 151; two-way ANOVA, ***P < 0.0001; followed by Bonferroni *post hoc* test ###*P* < 0.05). (D) The calcium response to H_2_O_2_ ± PF-5105679 is expressed as percentage of the maximum response represented by ionomycin (Student’s *t*-test, ***P < 0.0001). (E) Percentage of responding cells to H_2_O_2_ ± PF-5105679 (Student’s *t*-test, ^ns^*P* > 0.05).

Urothelial tissues from young mice were exposed to 0.003% H_2_O_2_ (*N* = 5) or Hepes buffer (*N* = 7) for 5 h, and qRT–PCR for TRPA1, TRPV1, TRPC4, TRPC5, TRPC6, TRPM2, TRPM4 and TRPM8 was performed. Of all the TRP channels examined, TRPM8 mRNA showed the greatest fold change level (3.4-fold change) in tissues treated with H_2_O_2_, compared with tissues treated with vehicle. A smaller upregulation was also found for TRPA1 and TRPM4 mRNA levels (< 2-fold change; Fig. 3A). TRPM8 protein was detected using Western blot analysis. Higher TRPM8 protein levels were found in the H_2_O_2-_treated tissues (*N* = 5) compared with the untreated (saline) tissues (*N* = 4) where TRPM8 was not detectable (Fig. 3B). As nonspecific bands were detected, LNCaP prostate cancer cell line was used as TRPM8-positive control to assess the right band size, and β-actin was used as loading control (Fig. 3B).

In calcium imaging experiments, urothelial cells isolated from young mice were exposed to 1 μm TRPM8 antagonist PF-5105679 (*N* = 4, *n* = 224) in the presence or absence of 0.003% H_2_O_2_, and a significant attenuation of the response to H_2_O_2_ was observed (*N* = 4, *n* = 151) without affecting the percentage of responding cells (Fig. 3C, two-way ANOVA, ***P < 0.0001; followed by Bonferroni *post hoc* test ###*P* < 0.05; Fig. 3D,E, Student’s *t*-test ***P < 0.0001 and ^ns^*P* = 0.06).

### Increase in oxidative stress markers in the urothelium as a result of aging

**Figure 4:**
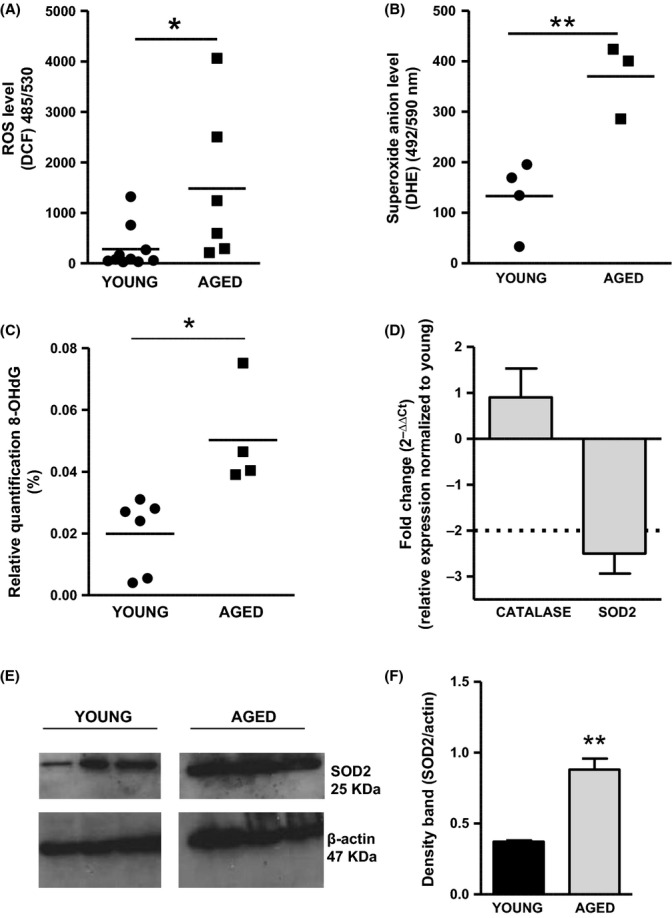
Detection of markers for oxidative stress in the urothelium in young and aged mice. (A) Higher intracellular reactive oxygen species (ROS) and (B) superoxide anion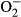 level in urothelial cells were found in aged mice compared with the young group (Student’s *t*-test, *P = 0.03; **P = 0.007). (C) Higher 8-OHdG level in DNA samples was detected in aged urothelium compared with the young group (Student’s *t*-test, *P = 0.01). (D) qRT-PCR for catalase and superoxide dismutase2 (SOD2) mRNA expression in urothelium from young and aged mice (*N* = 5). Results are expressed as fold change (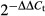) of aged compared with young mice and show a downregulation of SOD2 mRNA in aging. (E) Western blot for SOD2 protein level in the urothelium from young and aged mice (*N* = 3, each lane represents a different mouse). Results show a significant upregulation of SOD2 protein in aging (Student’s *t*-test, *P = 0.002). β-actin was used as loading control.

A significant increase in the intracellular ROS and superoxide anion 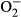 levels was found in the urothelial cells isolated from aged mice (*N* = 6, *N* = 3) compared with young mice (*N* = 10, *N* = 4; Fig. 4A,B, Student’s *t*-test, *P = 0.03; **P = 0.007). It is well established that DNA oxidation leads to more than 20 base modifications, the products of which (such as 8-hydroxy-deoxyguanosine, 8-OHdG) can be used to assess the level of oxidative DNA damage. In this study, higher levels of 8-OHdG were found in urothelial tissues as a result of aging (*N* = 4) compared with tissues from young mice (*N* = 6; Fig. 4C, Student’s *t*-test, *P = 0.01). qRT–PCR showed a downregulation of the antioxidant SOD2 but no change in catalase (CAT) mRNA level (Fig. 4D, *N* = 5). Western blot analysis found a higher level of SOD2 protein in the urothelium from aged mice compared with the young group (Fig. 4E,F, *N* = 3 Students *T*-test, **P = 0.002).

### TRPM8 is expressed in the urothelium as a result of aging, and it is modulated by the antioxidant NAC

**Figure 5:**
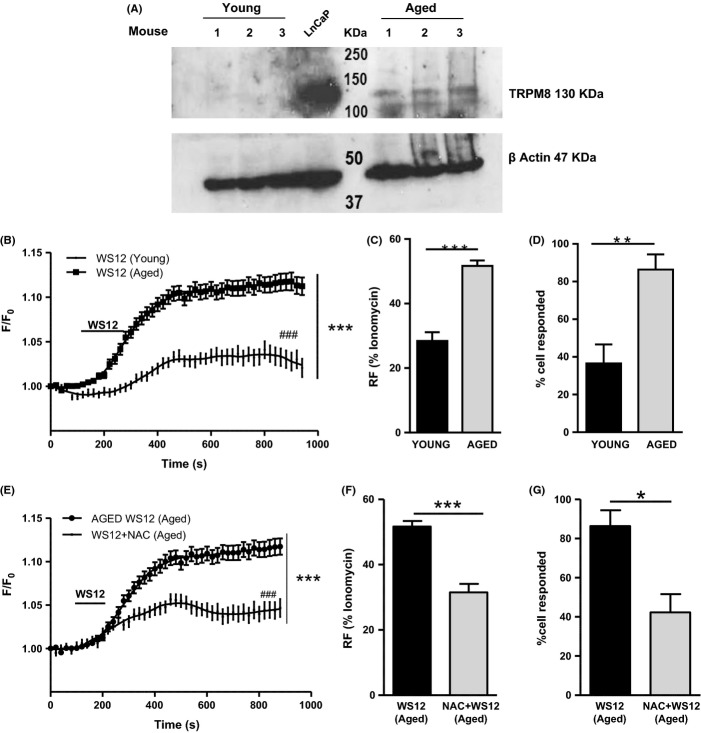
TRPM8 is expressed and is functional in the aged urothelium, and it is modulated by the reactive oxygen species (ROS) scavenger *N*-acetyl-cysteine (NAC). (A) Western blot analysis for TRPM8 protein level in the urothelium tissue from young and aged mice (*N* = 3, each lane represents a different mouse). Results show that TRPM8 is not detectable in the young group, while it is upregulated in aging. β-actin is used as loading control, and LNCaP cell line was used as positive control for TRPM8 expression. (B) Calcium imaging data on urothelial cells show a greater response to TRPM8 agonist WS12 in aging (*N* = 6, *n* = 209) compared with young mice (*N* = 5, *n* = 126; two-way ANOVA, ***P < 0.0001; followed by Bonferroni, ###*P* < 0.05). (C) The calcium response to WS12 of young and aged urothelial cells is expressed as percentage of the maximum ionomycin response. Results show that in aging, the response to WS12 is greater than in young group (Student’s *t*-test, ***P < 0.0001). (D) Representation of the percentage of responding cells to WS12 shows that in aging a higher number of urothelial cells responded to WS12 (Student’s *t*-test, **P = 0.003). (E) Calcium imaging data on aged urothelial cells treated with WS12 with (*N* = 4, *n* = 176) or without (*N* = 6, *n* = 209) 1 h preincubation with the antioxidant *N*-acetyl-cysteine (NAC) show that in the presence of NAC calcium response to WS12 is significantly attenuated (two-way ANOVA, ***P < 0.0001; followed by Bonferroni *post hoc* test, ^###^*P* < 0.05). (F) The calcium response to WS12 ± NAC is expressed as percentage of the maximum ionomycin response. Results show that NAC reduced the calcium response to WS12 (Student’s *t*-test, ***P < 0.0001). (G) Representation of the percentage of responding cells to WS12 ± NAC showing a decrease in responding cells in the presence of NAC (Student’s *t*-test, *P = 0.007).

Western blot for TRPM8 protein was performed in young and aged urothelial tissues (*N* = 3). LNCaP prostate cancer cell line was used as TRPM8-positive control to assess the exact band size, and β-actin was used as loading control. TRPM8 was found only in the urothelium from aged mice (Fig. 5A).

To investigate the function of TRPM8 in the urothelium, the calcium response to the TRPM8 agonist WS12 (1 μm) was evaluated in the urothelial cells from young (*N* = 5, *n* = 126) and aged mice (*N* = 6, *n* = 209; Fig. 5B, two-way ANOVA, ***P < 0.0001; followed by Bonferroni, ###*P* < 0.05). A greater calcium response was found in urothelial cells from aged mice (Fig. 5C, Student’s *t*-test, ***P < 0.0001) along with a higher percentage of responding cells (86%) compared with cells from young mice (36%) (Fig. 5D, Student’s *t*-test, **P = 0.003). Moreover, the response to WS12 in urothelial cells from aged mice was significantly attenuated by the preincubation with NAC, and moreover, the percentage of responding cells was significantly reduced (Fig. 5E, two-way ANOVA, ***P < 0.0001; followed by Bonferroni *post hoc* test, ###*P* < 0.05); Fig. 5F,G, Student’s *t*-test, **P = 0.003; ***P < 0.0001; *N* = 4, *n* = 176).

## Discussion

The mechanisms underlying the complex process of aging are still poorly understood; however, evidence suggests that accumulation of ROS can be one of the primary determinants of aging, leading to functional alterations and pathological conditions (Kregel & Zhang, 2007). Lower urinary tract is very susceptible to aging; in fact, bladder conditions, including OAB syndrome and its associated UI, are prevalent in the elderly. The epithelial lining of the bladder, known as the urothelium, closely interacts with the underlying sensory innervation, forming a urothelial sensory network which seems to play a key role in normal bladder physiology. It has been suggested that an alteration in this signalling network, either at the level of sensory nerve terminal or within the urothelium, may be a contributing factor in age-related pathological conditions (Birder *et al*., 2012). In our previous study, we reported significant changes in bladder function as a result of aging, which were indicative of bladder hypersensitivity and hyperactivity; moreover, an increase in urothelial cell excitability and transmitter release was found, supporting the hypothesis that the urothelial sensory signalling is altered as a result of aging (Daly *et al*., 2014). Another experimental study on aged rodents suggests that aging impairs contractile response in the bladder, and this effect correlates with an increase in oxidative stress markers, suggesting that oxidative stress may contribute to alter bladder function in aging (Gomez-Pinilla *et al*., 2007b). However, the mechanisms underlying the effect of oxidative stress on bladder physiology are still unclear.

A study performed by Masuda *et al*. (2008) showed that intravesical administration of H_2_O_2_ impaired detrusor contractility, and this response was attenuated by capsaicin; the authors therefore interpreted that oxidative stress may alter capsaicin-sensitive c-fibre sensitivity. However, to our knowledge, there is no direct physiological evidence showing that oxidative stress stimulates bladder sensory signalling, and the mechanisms underlying this sensory transduction remain unclear.

### Induction of oxidative stress causes alterations in urothelial sensory signalling

In the present study, *in vitro* extracellular nerve recordings were performed to investigate whether an acute oxidative stress stimulus (induced by application of H_2_O_2_) was able to cause global functional changes on the bladder sensory signalling and detrusor contractility. Our data show that extravesical application of 0.003% and 0.03% H_2_O_2_ causes a dose-dependent increase in bladder afferent nerve activity concurrent with a moderate increase in bladder pressure (4–5 mmHg) indicative of muscle contraction. Strikingly, the afferent response was recoverable after only a few minutes, but the changes in pressure were not readily reversible. This may be due to a different time courses between afferent and muscle components; however, the underlying mechanisms are not well defined.

As mechanosensitive afferents respond to contraction and changes in muscle tone, and some of the afferent response may be secondary to the increase in intravesical pressure; however, the observed increase in nerve activity was disproportionately high compared with such a moderate increase in intravesical pressure, suggesting that some of the afferent response may also be driven by mechanisms independent of the muscle. Studies from Xu and Gebhart (2008) and Zagorodnyuk *et al*. (2007) clearly show that sensory innervation of the bladder arises not only from the detrusor muscle but also from the serosal, urothelial and suburothelial layers. Interestingly, when H_2_O_2_ was applied intraluminally, only the highest concentration (0.03%) produced an increase in afferent firing, whereas no effect was seen with the lowest concentration (0.003%). The lack of effect observed with the lowest concentration of H_2_O_2_ in the extra- and intravesical application is likely to reflect the effective barrier function of the urothelium, which acts to prevent luminal agents from crossing through the bladder wall. As in the extravesical application there is no protective diffusion barrier, H_2_O_2_ can freely act on the serosal and muscle layers of the bladder inducing increase in nerve activity at both concentration. In the light of these findings, even though the functional changes that we observed in response to H_2_O_2_ are likely to be driven by multiple converging components (such as detrusor muscle cells, interstitial cells, sensory and motor nerve terminals, intramural ganglia and the urothelium), it is tempting to speculate that part of the H_2_O_2_ responses arises due to activation of chemosensitive afferents innervating the urothelium/suburothelium. To specifically look at the functional changes on the urothelium as a result of an acute oxidative stress stimulus, calcium imaging experiments were then conducted on isolated urothelial cells exposed to subtoxic concentrations of H_2_O_2_. Our data clearly show that an acute oxidative stress stimulus dramatically increased intracellular calcium in the urothelial cells, and these responses were attenuated by the preincubation with the ROS scavenger NAC. All together, these data support the hypothesis that an acute oxidative stress is able to induce functional changes in the urothelial sensory signalling and suggest that the urothelium is susceptible to oxidative stress, with possible implications for aging. Although the downstream signalling mechanisms that occur as a result of these responses remain to be elucidated, changes in urothelial cell activity/excitability could lead to an alteration in transmitter release from the urothelium and in turn changes in afferent excitability.

### Oxidative status in the urothelium changes as a result of aging

Accumulating evidence suggests that aging is associated with a higher generation of ROS, resulting in a progressive oxidation of nucleic acid, proteins and lipids (Droge & Schipper, 2007). These harmful oxidative conditions can lead to functional changes, contributing to increase the susceptibility to a variety of diseases. To date, the effect of oxidative stress and aging on the urothelium has received little research attention. A recent study by Perse *et al*. demonstrated that the urothelium is specifically altered by oxidative changes in 20-month-old female mice, undergoing impairment of cellular structures, such as lysosomes and mitochondria. The authors also found a general increase in the activity of antioxidant enzymes, but these were unable to prevent the observed oxidative damage in the urothelium (Perse *et al*., 2013). Accumulating oxidative DNA damage in aging has been reported in several aged tissues including liver, heart, kidney, skeletal muscle, spleen and brain, in rodents (Hamilton *et al*., 2001) and human (Gianni *et al*., 2004; Short *et al*., 2005). In line with these previous findings, the present study suggests that aging is correlated with an imbalance between oxidants and antioxidants in the aged mouse urothelium and that an accumulation of ROS may enhance urothelial sensory signalling. In this study, we show a significant increase in oxidative stress markers including intracellular ROS, 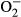 and 8-OHdG levels in the urothelium as a result of aging, suggesting also a greater oxidative damage to DNA compared with young mice. Moreover, we found a downregulation of the antioxidant SOD2 mRNA that was concurrent with a significant upregulation of SOD2 protein in the urothelium from aged mice. There are a number of reasons that may account for this apparent discrepancy: firstly, a compromised regulation of this enzyme could be responsible for the higher intracellular superoxide anion levels detected in the urothelial cells as a result of aging. Secondly, it is important to take into account that our urothelial/suburothelial tissues might also contain other cell types such as blood vessels and nerve terminals that may have contributed to the overall SOD2 protein expression. Thirdly, there are studies in the literature that suggest that mRNA and protein expression can be inversely related. In many cases, this has been shown to be due to multiple regulatory mechanisms occurring after mRNAs production, speed of transcription/translation and protein stability (Vogel & Marcotte, 2012).

### Oxidative stress and aging activate TRPM8 in the urothelium

Previous studies have suggested that TRP channels such as TRPC3, TRPC4, TRPM2 and TRPM7 may be gated by oxidative stress, to mediate calcium influx and leading eventually to tissue damage, pathophysiological conditions or cell death (Miller, 2006; Takahashi *et al*., 2011; Naziroglu, 2012). In the present study, urothelial expression of several TRP channels mRNA was assessed after an acute induction of oxidative stress. Of all the genes tested, TRPM8 showed the greatest fold change following H_2_O_2_ treatment, suggesting that oxidative stress may modulate TRPM8 expression. Treatment with H_2_O_2_ also induced a smaller upregulation of TRPA1 and TRPM4 (fold change < 2), suggesting that the expression of other TRP channels may be altered by oxidative stress as indeed suggested by studies in literature and may warrant further study.

The effect of oxidative stress on TRPM8 expression was evaluated at the protein level where an upregulation of this receptor after treatment with H_2_O_2_ was observed, supporting the hypothesis that TRPM8 expression is regulated by oxidative stress. In absence of any treatment, a very low expression of TRPM8 was found in the urothelium. This is in line with previous studies from Everaerts *et al*. (2009) and Yu *et al*. (2010), who showed very low expression of TRPM8 in urothelial tissue. Moreover, TRPM8 antibody showed also nonselective bindings (resulting in nonspecific bands) but only in the samples treated with H_2_O_2_. This may depend on the treatment with H_2_O_2_ itself, which can alter the protein expression profile in the urothelium, inducing, for example, an upregulation of certain proteins or post-translational modifications such as protein phosphorylation or production of dimeric proteins, which can bind the TRPM8 antibody in a nonspecific manner. Moreover, in our calcium imaging experiments, the urothelial calcium response to H_2_0_2_ was significantly attenuated by the selective TRPM8 antagonist PF-5105679, suggesting for the first time that TRPM8 receptors may be involved in altering the calcium homeostasis induced by oxidative stress in the urothelial cells. The reason for the existence of the ‘cold sensing’ receptor TRPM8 in the bladder is still unknown. Previous studies have shown a positive correlation between TRPM8 expression and voiding frequency in patients with bladder conditions, such as OAB symptoms and painful bladder syndrome (PBS) patients (Mukerji *et al*., 2006b) and an involvement of this channel on micturition reflex and nociceptive signalling (Lashinger *et al*., 2008). This study suggests that TRPM8 may function as a ‘sensor’, which can be activated in all those detrimental conditions characterized by ROS generation, such as inflammation, hypoxia or aging. To further support this idea, we investigated the protein expression of TRPM8 and its function in the urothelium from aged mice. Interestingly, a greater TRPM8 protein expression was found in the urothelium from aged mice compared with the young mice, where we were not able to detect any TRPM8 protein expression. Nonspecific bands were also observed but only in the aged samples. As we hypothesized for the treatment with H_2_O_2_, age-related changes in the protein expression profile may occur, including upregulation of other proteins and post-translational modifications, which can cause a nonselective binding to the TRPM8 antibody as a result of aging. The upregulation of TRPM8 protein observed in aged urothelium was also concurrent with an enhanced function of this receptor, confirmed by an increased calcium response to the TRPM8 agonist WS12. Interestingly, the treatment with the ROS scavenger NAC significantly reduced the WS12 response in aged urothelial cells, suggesting that high level of ROS occurring in aging may cause TRPM8 activation. A possible regulation of TRP channels by antioxidants has been previously proposed. Naziroglu (2012) observed that oxidative stress-induced activation of TRPM2, TRPC3, TRPC5 and TRPV1 in neuronal cells and these receptors were modulated by CAT, suggesting antioxidant-dependent activation/inhibition of the channels. The data from our study would now also suggest that TRPM8 may be among these ion channels. Surprisingly, a very small calcium response to WS12 was also found in a low percentage of urothelial cells from young animals, where TRPM8 protein was not detected. This may be due to a nonspecific effect of the agonist [although, studies from Ma *et al*. (2008) and from our laboratory, WS12 has been shown to be a very selective agonist for TRPM8] or the presence of a really small amount of TRPM8 protein, which was below the detection level for Western blot. The observation of this small calcium response to WS12 in young urothelial cells is in contrast to previous studies, which found no response to TRPM8 stimulation using isolated urothelial cells (Everaerts *et al*., 2009). However, some key experimental differences such as differences in which agonists were applied (icilin and menthol vs. WS12), positive controls used (ATP vs. ionomycin) and thresholds used for the analysis (i.e. what was considered as a responding cell), could explain this discrepancy.

The upregulation of TRPM8 in H_2_O_2_-treated and in aged tissues, as well as the increased level of oxidative stress markers found in aged urothelium, supports the hypothesis that TRPM8 may be modulated by age-related oxidative stress. Although an acute induction of oxidative stress (such as that obtained with a short-term treatment with H_2_O_2_) cannot be fully representative of what really happens in aging, these findings suggest that an induced (H_2_O_2_) and a physiological occurring oxidative stress (aging) may have some similar effects. Moreover, although this study looks specifically at the bladder urothelium, many other visceral organs have a similar epithelial lining. This raises the exciting possibility that the mechanisms described in this study could represent a universal action of TRPM8 in sensing oxidative stress and functional changes that occur as a result of aging. It has already been well established that TRPM8 is increased in age-associated malignancies such as colon and prostate cancer but whether expression of TRPM8 is increased purely as a result of aging has yet to be established. Of course at this time, this is the only speculation, and more studies are still required to investigate the physiological role of TRPM8 throughout the body.

In conclusion, this study suggests a novel role of TRPM8 as a ‘sensor’, which can be activated in detrimental conditions characterized by ROS generation, such as inflammation, hypoxia or aging. The upregulation of TRPM8 in aged mice bladder confirms this hypothesis and suggests a potential role of this receptor in altering urothelial sensory function in aging. If true, this could have exciting implications for understanding the underlying aetiology of bladder symptoms such as OAB, which are strongly associated with advancing age. This may highlight a potential co-adjuvant therapeutic strategy for the prevention and treatment of this type of bladder dysfunction. However, the therapeutic potential of TRPM8 and antioxidants to provide new targets for bladder pharmacotherapy needs further translational study in humans.

## Experimental procedures

### Animals

All experiments were conducted according to the University of Sheffield’s Animal Care Committee, under an approved UK protocol and project licence. Five-month-old (young) and 24-month-old (aged) C57/BL6 male mice were used in this study (Harlan and Charles River UK). Animals were sacrificed by cervical dislocation after anaesthesia with isoflurane.

### *In vitro* extracellular nerve recording

Bladder nerve recording was conducted using an *in vitro* model previously described (see Daly *et al*., 2007 and Collins *et al*., 2013 for further details). The whole pelvic region from young mice (*N* = 6) was dissected and continually perfused with gassed (95% O_2_, 5% CO_2_) Krebs–bicarbonate solution (mm: NaCl 118.4, NaHCO_3_ 24.9, CaCl_2_ 1.9, MgSO_4_ 1.2, KH_2_PO_4_ 1.2, glucose 11.7) and maintained at 35 °C (to prevent rapid degradation of the tissue). The urethra and the dome were catheterized allowing recording of intravesical pressure. A mixed population of pelvic and hypogastric nerves was dissected and placed into a glass electrode for recording. The electrical activity was recorded, amplified, filtered and captured by a computer processor that counts number of action potentials crossing a preset threshold via a power 1401 interface and Spike2 software (version 7; Cambridge electronic design, Cambridge, UK). For the extravesical application, bladder was kept under isovolumetric condition (15 mmHg) and 0.003% and 0.03% g/v H_2_O_2_ (Sigma, St. Louis, MO, USA) were applied for 2 min, leaving 20 min before the following application. For the intravesical application, 0.003% and 0.03% H_2_O_2_ were continuously perfused into the bladder for 30 min. Results are expressed as maximum afferent firing as mean impulses per second or changes in intravesical pressure (mmHg) compared with baseline or saline.

### Primary urothelial cell culture

The entire bladder from young and aged mice was cut longitudinally from the urethra and pinned flat with the urothelium on the top surface. Urothelial cells were cultured as previously described (Everaerts *et al*., 2009). The cell suspension was counted and plated on collagen (IV)-coated coverslips or 96-wells.

### Cell viability assay

Cell viability was evaluated by the MTT method. Urothelial cells from young mice were plated as described above, at a density of 1 × 10^4^ per well. The cells were treated with 0.003% H_2_O_2_ for 10 min, and then, 3-4,5-dimethylthiazol-2-yl)-2,5-diphenyltetrazoliumbromide (MTT; Sigma) was added in the media, and finally, the cells were incubated at 37 °C for 4 h. After removing the media, 100 μL of isopropanol was added to dissolve the crystals. Absorbance was read at 550 nm in a plate reader. Results are expressed as percentage relative to the untreated cells, set as 100%.

### Calcium imaging of cultured mouse urothelial cells

Urothelial cells (20–24 h) from young and aged mice were loaded with 2 μm fura-2acetoxymethyil (fura-2AM) for 30 min and washed in Hepes buffer. Cells from young mice were stimulated with H_2_O_2_ 0.003% ± NAC (10 mm; Sigma) or ± PF-5105679 (1 μm; Pfizer, Sandwich, UK) and WS12 (1 μm). Cells from aged mice were stimulated with WS12 (1 μm) ± NAC. Ionomycin (5 μm) was applied as positive control, and changes in intracellular calcium [Ca^2+^]_i_ were monitored in real time. [Ca^2+^]_i_ was calculated as ratio between the fluorescence signal at 340 and 380 nm for the responding cells. The ratio analysis was then converted into *F*/*F*_0_ representing a normalization of the fluorescence readings during drug application (*F*) to the reading at the baseline (*F*_0_). Results are also expressed as percentage to ionomycin response, which is unchanged in each group. All the drugs were prepared in Hepes buffer.

### Intracellular ROS and superoxide anion (O^2-^) detection

1 × 10^4^ urothelial cells from young and aged mice were plated in a collagen-coated 96-well plate. The cells were incubated for 30 min with 40 μm 2,7-dichlorofluorescein diacetate (DCFDA; Sigma) and 20 μm dihydroethidium (DHE; Life Technologies Ltd, Paisley, UK) for the intracellular ROS and superoxide anion levels. Intracellular ROS level was also measured in the urothelial cells from young mice, with or without 10 min treatment with 0.003% H_2_O_2_. The fluorescence was read at 485/530 nm (ex/em) for DCFDA (Wang & Joseph, 1999) and 492 nm/590 nm for DHE, and results are expressed as relative fluorescence unit.

### Urothelium/suburothelium tissue dissection

The bladder from young and aged mice was cut as described above. Urothelium/suburothelium was dissected from the detrusor muscle under the microscope and kept in RNA later or snap frozen and stored at −80 °C until use.

### 8-hydroxydeoxyguanosine (8-OHdG) level detection

DNA from young and aged urothelial tissues was extracted using DNeasy blood and tissue kit (Qiagen, Manchester, UK) and quantified with Nanodrop (Thermo Scientific, Wilmington, DE, USA). The level of 8-hydroxy-2′-deoxiguanosine (8-OHdG) was measured using EpiQuick 8-OHdG DNA damage quantification direct kit (Epigentek, Cambridge Bioscience Ltd, Cambridge, UK) and quantified by fluorescence at 530ex/590em nm. The results are expressed as relative quantification (%) to a positive control provided by the kit and normalized to the input DNA (ng), using the recommended formula.

### Quantitative real time PCR (qRT–PCR)

Urothelium/suburothelium tissues from young and aged mice were used for CAT and superoxide dismutase 2 (SOD2) gene expression analysis. Transient receptor potential channels’ mRNA level analysis was performed using control tissues kept for 5 h in Hepes buffer (Hepes 10 mm, NaCl 135 mm, KCl 5 mm, glucose 10 mm, CaCl2 2 mm and MgCl_2_ 1 mm) pH7.4 at 36.5 °C with or without H_2_O_2_ 0.003%. Total RNA was extracted and reverse transcribed using RNeasy mini Kit (Qiagen) and High-Capacity cDNA Reverse Transcription kit (Applied Biosystems, Life Technologies Ltd, Paisley, UK). qPCR was performed using TaqMan primer/probe mix (IDT) for CAT and SOD2 and for TRPA1, TRPV1, TRPC4, TRPC5, TRPC6, TRPM2, TRPM4 and TRPM8. GAPDH was used as reference gene. The results are expressed as fold change (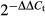; Livak & Schmittgen, 2001).

### Western blot

Urothelium sub urothelium tissues dissected from control and aged bladders and from untreated (saline) and H_2_O_2_-treated control bladders were homogenized in Ripa Buffer (Sigma) containing protease inhibitors. For all Western blot experiments, the proteins were extracted from the whole homogenates, without separating the membrane fraction. The protein quantification was assessed using a Bradford assay (Bio-Rad Laboratories Ltd, Hertfordshire, UK): a standard curve was created using known concentration of bovine serum albumin, and it was run together with the unknown lysates. Using a plate reader set at 595 nm, the absorbance was read and the protein concentration was extrapolated from the standard curve. Ten microgram protein was resolved in 8% SDS-PAGE, transferred to nitrocellulose membrane (Protran; VWR Lutterworth, Leicestershire, UK) and incubated with anti-TRPM8 (1:1000), anti-SOD2 (1:5000) and ant-β-actin (1:1000; AbCam, Cambridge, UK). LNCaP prostate cancer cell line was used as a positive control for TRPM8 expression. Band intensities were analysed using ImageJ software (download freely available), and results are expressed as ratio, using β-actin as loading control.

### Data analysis

Data are expressed as mean ± SEM. Statistical analysis was performed using Student’s *t*-test or one-way ANOVA or two-way ANOVA followed by the appropriate *post hoc* test. Significance was confirmed at *P* < 0.05. All the statistical analyses were performed using GraphPad Prism 5 (GraphPad Software Inc., La Jolla, CA, USA).
